# Association of Changes in Missouri Firearm Laws With Adolescent and Young Adult Suicides by Firearms

**DOI:** 10.1001/jamanetworkopen.2020.24303

**Published:** 2020-11-04

**Authors:** Apurva Bhatt, Xi Wang, An-Lin Cheng, Kalee L. Morris, Luke Beyer, Abbie Chestnut, Kristy Steigerwalt, Jeffrey Metzner

**Affiliations:** 1 University of Missouri-Kansas City/Center for Behavioral Medicine, Kansas City; 2 Truman Medical Centers, Kansas City, Missouri; 3 Department of Biomedical and Health Informatics, University of Missouri-Kansas City, Kansas City; 4 University of Missouri-Kansas City School of Medicine, Kansas City; 5 Kansas City University of Medicine and Biosciences, Kansas City, Missouri

## Abstract

**Importance:**

Suicide by firearm is a major cause of mortality in young Missouri residents. Changes in statewide firearm policy may have contributed to changes in suicide rates.

**Objective:**

To evaluate if changes in Missouri permit-to-purchase (PTP) and concealed carry firearm laws were associated with changes in rates of suicide by firearms in young Missouri residents.

**Design, Settings, and Participants:**

This cross-sectional study examined rates of suicide by firearm in Missouri among adolescents (ages 14-18 years) and young adults (ages 19-24 years) from January 1999 to December 2018, following changes to state PTP and concealed carry law, in comparison with a donor state pool with existing firearm laws (13 states in PTP pool; 42 states in concealed carry pool) that did not make changes during this period. This study used a quasiexperimental, synthetic control design at the state level that defined Missouri as the treated state and treatment as changes in firearm laws.

**Exposures:**

Legislative changes to Missouri’s PTP and concealed carry firearm laws.

**Main Outcomes and Measures:**

Age-adjusted annual rates of firearm-related suicide mortality per 100 000 people for adolescents (aged 14-18 years) and young adults (aged 19-24 years).

**Results:**

Repeal of the PTP law was associated with a 21.8% increase in firearm suicide rates in young adults aged 19 to 24 years in Missouri. Lowering the minimum age of concealed carry to 19 years in Missouri was associated with a 32.0% increase in firearm suicide rates and a 29.7% increase in nonfirearm suicide rates in adolescents aged 14 to 18 years, and a 7.2% increase in firearm suicide rates in young adults aged 19 to 24 years.

**Conclusions and Relevance:**

Increases in rates of suicide by firearms in adolescents and young adults were seen following repeal of Missouri’s PTP law and lowering of the minimum age for concealed carry in 2014. Changes in Missouri’s firearm policies may be an important contributor to rates of suicide by firearm in young Missouri residents.

## Key Points

**Question:**

Were changes in Missouri’s firearm laws associated with rates of suicide by firearms in young Missouri residents?

**Findings:**

In this cross-sectional study, repeal of the permit-to-purchase law was associated with a 21.8% increase in firearm suicide rates in young adults aged 19 to 24 years in Missouri. Lowering the minimum age of concealed carry to age 19 years in Missouri was associated with increased firearm suicide rates by 32.0% and nonfirearm suicide rates by 29.7% in adolescents aged 14 to 18 years, and increased firearm suicide rates by 7.2% in young adults aged 19 to 24 years.

**Meaning:**

These findings suggest that changes in Missouri’s permit-to-purchase and concealed carry firearm laws may have contributed to increased rates of firearm suicides in young Missouri residents.

## Teaser

This cross-sectional study examines changes in firearm suicide rates in Missouri following the loosening of state firearm restrictions by comparing reported suicide rates with states with similar demographic characteristics that did not change firearm laws.

## Introduction

Youth suicide by firearm is a difficult occurrence faced in health care. In 2017, the US Center for Disease Control and Prevention (CDC) reported that suicide was the second leading cause of death for children and young adults aged 10 to 24 years.^1^ In 2017, Missouri ranked sixth in the nation in firearm deaths.^2^ Firearm-related deaths are the second leading cause of death in Missouri children aged 1 to 17 years.^3^ In Missouri, 58% of firearm deaths are suicides and more than 58% of suicide deaths involve firearms.^3^ In 2018, every 4 days a young person in Missouri died by suicide via firearm.^4^

It is important to be cognizant of the current state of affairs regarding Missouri’s firearm policies, which include some of the least restrictive gun laws in the US. Currently in Missouri, private possession of firearms is permitted without a license.^5,6^ It is unlawful to sell or give a firearm to anyone under the age of 18 without parental consent.^7,8^ While federal law requires licensed firearms dealers to run background checks, buyers of firearms in private sales are not required to pass official background checks before taking possession of the firearm.^9^ There is no established waiting period for a firearm purchase to be completed.^8^ Current regulations do not have written specifications for safe storage of private firearms and ammunition.^10^

Missouri recently weakened several firearm policies. In 2007, Missouri removed a requirement for gun owners to have a state permit-to-purchase (PTP) license for concealable firearms. The PTP law (enacted in 1981) required all handgun purchasers to have a valid PTP license to purchase a handgun from any seller, licensed or unlicensed. Residents were required to submit a PTP application to their local sheriff’s office, which verified the accuracy of statements made in the application. The application required basic information (ie, name, age, and reason for desiring the permit) and that the applicant (1) was at least 21 years old; (2) had not pled guilty to, been convicted of, or was currently charged with a crime punishable by imprisonment for over 1 year; (3) was not a fugitive from justice; (4) had not been dishonorably discharged from the US military; (5) was not known to be habitually in an intoxicated or drugged condition; and (6) was not currently adjudged mentally incompetent and had not been committed to a mental health facility.^5^

In 2011, Missouri lowered the legal age to obtain a concealed carry permit from 23 to 21 years,^11,12^ and further reduced it to age 19 in 2014.^13^ Finally, in 2017, a law was enacted that allowed for permitless carry for all gun owners in most public places. Currently in Missouri, any person at least age 19 years may carry a firearm in plain view (ie, open carry) or in hidden view (concealed carry) in most places without a permit.^5,14^

Prior studies have been published regarding firearms and youth suicide rate. A 2019 study^15^ reported that “for each 10 percentage-point increase in household gun ownership, the youth suicide rate increased by 26.9%.” Additional research demonstrated that 82% of firearm-related suicides among youth involved a firearm owned by a household member.^15^ The objective of our study was to evaluate the association of the repeal of Missouri’s PTP law and changes in concealed carry law with firearm-related suicide rates in young Missouri residents ages 14 to 24 years.

## Methods

### Design and Study Sample

In this cross-sectional study, we conducted separate analyses on 2 Missouri firearm law changes and firearm suicide rates among youth (between ages 14 and 18 years) and young adults (between ages 19 and 24 years) using a quasiexperimental, synthetic control design at the state level, with Missouri as the treated state and treatment defined as a change in Missouri firearm law. The main analysis considered the preintervention period to be all years before a firearm law change for which data were available and assessed outcomes associated with the years postintervention (or until the next law change). We analyzed the repeal of Missouri’s PTP law in 2007, with the preintervention period being from January 1999 to December 2006, and the postintervention period being from January 2007 to December 2010. The second law evaluated was the reduction in the concealed carry permit age requirement, which had 2 changes (in 2011 and 2014). For this reason, the concealed carry study period was divided into pre-first (2008-2010), post-first (January 2011 to December 2013), and the post-second period (January 2014 to December 2018). Data were analyzed from January 2014 to December 2018. Because this study included only fatalities, and all data was obtained from publicly available sources, it did not meet the definition of human subject research and was deemed exempt from informed consent requirements and institutional review board approval by the University of Missouri-Kansas City.

### Participants

The potential control states for the PTP analysis, also known as the donor pool, were states that had an existing PTP law and no change in that law during the study period (January 1999 to December 2010). Legal research was completed using Westlaw,^16^ a software providing access to all state and federal laws. Because there is no published analysis providing comparability between all US state laws, each state law was reviewed individually and assessed for comparability. Control states for the PTP analysis were Connecticut, Hawaii, Kansas, Illinois, Iowa, Massachusetts, Michigan, Minnesota, Nebraska, New Jersey, New York, North Carolina, and Rhode Island. For the concealed carry analysis, states were only included in the donor pool if they had an existing concealed carry law and no change in that law during the study period (January 2008 to December 2018). These states were Alabama, Alaska, Arizona, Arkansas, California, Colorado, Connecticut, Delaware, Florida, Georgia, Hawaii, Indiana, Iowa, Kansas, Kentucky, Louisiana, Maine, Massachusetts, Michigan, Minnesota, Mississippi, Montana, Nebraska, Nevada, New Hampshire, New Jersey, New Mexico, New York, North Carolina, Ohio, Oklahoma, Oregon, Pennsylvania, Rhode Island, South Carolina, South Dakota, Tennessee, Texas, Utah, Washington, West Virginia, and Wyoming.

### Data Sources and Variables

#### Outcomes

The main outcomes were age-adjusted annual rates of firearm-related suicides per 100 000 people for adolescents (ages 14 to 18 years) and young adults (ages 19 to 24 years). We also included rates of nonfirearm suicides as an additional outcome (an outcome that should not be associated with polices that could increase access to firearms; if there was a correlation, it would likely be in the opposite direction [ie, nonfirearm methods would be substituted by firearms, lowering nonfirearm suicide rates]). An increase in nonfirearm suicide rates following firearm law changes would likely be the result of other unmeasured confounders.

State-level, age-adjusted annual rates of suicide by firearms and nonfirearm means were derived from death certificate data from the CDC Web-based Injury Statistics Query and Reporting Systems (WISQARS).^4^ Data was filtered to include death profiles from individuals between ages 14 and 18 years and 19 and 24 years between January 1999 and December 2018, both males and females of all races and ethnicities, with cause of death being intentional suicide by firearm and suicide by nonfirearm means. A disadvantage of using the WISQARS was that it suppressed data during years with very few suicides to protect data anonymity. We performed simple imputation using the mean of the entire study period for states with missing values. If a state had missing values for the entire study period completely, it was excluded from the donor pool for the corresponding subanalysis.

#### Covariates

For both age groups, we controlled for the following state-level factors, chosen because of their association with suicide risk in the published literature: (1) rurality (population density),^17,18,19^ (2) unemployment rates,^20^ (3) percentage of residents below the federal poverty line,^20^ (4) educational attainment,^20^ and (5) household gun ownership.^15^ State-level population density data was obtained from the US Census Bureau.^21^ Unemployment rates were obtained from the US Bureau of Labor Statistics.^22^ Percentage of residents below the poverty line and educational attainment (ie, receiving a college degree or higher) data were obtained from the American Community Survey^23^ and the US Census.^24^ Regarding household gun ownership, prior studies have used the ratio of firearm suicides to all suicides (FS/S) as a proxy for household firearm ownership.^15,25^ This proxy is highly correlated (*r* = 0.80) with state-specific measures of firearm ownership on a cross-sectional basis.^15,25^ For statewide firearm suicides and total suicides, we used age-adjusted rates from the WISQARS.

For the adolescent age group, we controlled for additional factors associated with youth suicide risk for which data was easily obtainable from the CDC Youth Risk Behavior Survey (YRBSS)^26^: (1) prevalence of severe negative affect (ie, in the past 12 months, having felt so sad or hopeless almost every day for 2 weeks or more that the young person stopped doing some usual activities); (2) seriously considered attempting suicide; (3) suicidal planning (having made a plan for a suicide attempt); and (4) suicide attempts. Regarding drug use, both marijuana use^27^ and heroin use^28^ have been associated with increased suicide risk, so data on these variables were obtained from the YRBSS (past 30-day marijuana use and prevalence of lifetime heroin use). Binge drinking has been associated with adolescent suicide attempts,^29^ so self-reported binge drinking behavior data was obtained, but data were only available for 2017. Three states in the donor pool did not participate in the YRBSS (Minnesota, Oregon, and Washington).

### Statistical Analysis

We used the synthetic control method^30,31,32^ to create a weighted combination of control states (ie, a synthetic Missouri) from the donor pool that best approximated the relevant characteristics of the treated state (ie, Missouri) prior to the law changes. The synthetic Missouri provided an estimate of expected firearm suicide rates in Missouri if the laws had not changed. The outcome associated with each policy was estimated as the difference between the values in the treated state and in the synthetic control group in the postintervention period. Consistent with other studies that have used this method,^32^ we determined the mean annual differences for the postintervention period according to the study design, which were 4 years after PTP repeal, 3 years after the 2011 concealed carry change, and 5 years after the 2014 concealed carry change.

Given that the synthetic control group method does not produce traditional measures of uncertainty (eg, 95% confidence intervals), inference is based on permutation tests, with the principle of alliteratively applying the synthetic control method by randomly reassigning the intervention across the control states to produce a set of placebo effects. The estimated change in suicide rates for Missouri can then be compared with the size of these other change estimates. The comparison is informative about the magnitude of the change associated with the intervention that was observed for Missouri.

We showed results produced by states with a comparable fit (root mean squared prediction error [RMSPE] less than 2 times the RMSPE for Missouri) in the preintervention period. We assessed whether the change in suicide rates estimated by the synthetic control method for the Missouri law changes is large relative to the change estimated for a state chosen at random using R Synth package version 3.6.1 (R Project for Statistical Computing).

## Results

From 1999 to 2009, firearm suicide rates in the adolescent group were steadily declining in Missouri, starting at 5.58 per 100 000 individuals (age-adjusted rate using a standard population of 2000) in 1999 down to 2.63 in 2009. From 2010 to 2018, firearm suicide rates in the adolescent group steadily increased up to 9.23 per 100 000 in 2018. Nonfirearm suicide rates remained relatively steady from 1999 to 2014 and peaked in 2018 (9.23 per 100 000 people). Annual trends in firearm and nonfirearm suicide rates for both analyses are shown in the eFigures 1, 2, and 3 in the Supplement.

From 1999 to 2007, firearm suicide rates were declining in the young adult group in Missouri. In 2008, there was an increase to 10.25 per 100 000 people, followed by a modest decline to 7.07 per 100 000 people in 2011. Another increase in firearm suicide rates was seen in 2012 (10.30 per 100 000 people), which was followed by a decrease until 2014 (7.36 per 100 000 people). From 2014 to 2017, rates of suicide by firearms have been increasing, peaking at 16.22 per 100 000 people in 2017. Nonfirearm suicide rates in the young adult group remained relatively stable until 2011.

### Results From the Synthetic Control Group Method

#### Repeal of PTP Law

**Figure 1.**
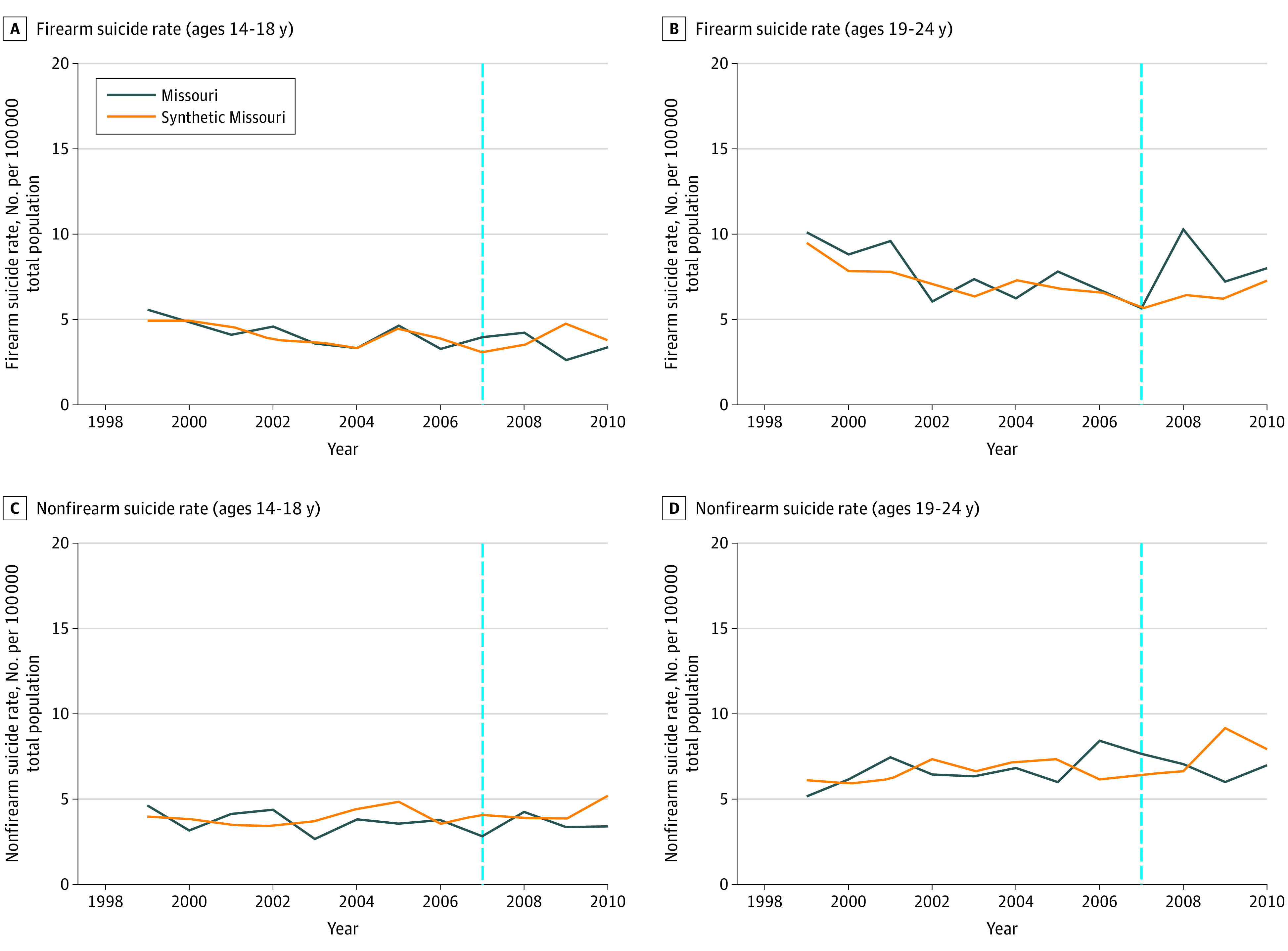
Trend in Annual Rate of Firearm and Nonfirearm Suicides per 100 000 People in Missouri and Synthetic Missouri Over the Permit-to-Purchase (PTP) Repeal Study Period The blue dotted line indicates the year the PTP repeal was passed in the Missouri state legislature. Synthetic Missouri is a weighted combination of control states that best approximated relevant characteristics of Missouri prior to the law changes examined by the study.

Of the 13 donor pool states, 11 had nonzero weights and were included in 1 or more of the synthetic controls for the 4 outcomes (eTable 1 in the Supplement). Trends for firearm suicide and nonfirearm suicide rates for the adolescent group in the preintervention period were similar for Missouri and synthetic Missouri (Figure 1). Trends in firearm suicide rates in the young adult group in the preintervention period were similar for Missouri and synthetic Missouri, although rates in Missouri were higher between 1999 and 2002 (Figure 1). Trends for nonfirearm suicide rates for the young adult group in the preintervention period were similar until 2006, when rates in Missouri were higher than synthetic Missouri (Figure 1).

Estimated absolute and relative associations between the change in PTP policy and each outcome and the results from the permutation tests are presented in the Table. We restricted the comparison states to those with a reasonable preintervention fit (≤2 times the RMSPE for Missouri). The mean difference in firearm suicide rates between Missouri and synthetic Missouri in the youth group in the postintervention period was −0.2 per 100 000 people, corresponding to a 5.2% decrease. For nonfirearm suicides, the difference in rate was −0.8 per 100 000 people, corresponding to a 19.0% decrease. The mean difference in rates of firearm suicides in the young adult group was 1.4 per 100 000 people, corresponding to a 21.8% increase. For nonfirearm suicides, the difference in rates was −0.6 per 100 000 people, corresponding to an 8.1% decrease.

**Table.** Association Between Permit-to-Purchase (PTP) Repeal and Changes of Concealed Carry Laws and Firearm and Nonfirearm Suicide Rates in Missouri for the Postintervention Periods

	Adolescent (ages 14-18 y)		Young adult (ages 19-24 y)	
	Firearm suicide	Nonfirearm suicide	Firearm suicide	Nonfirearm suicide
**PTP repeal**				
Missouri’s mean rate per 100 000 individuals after repeal	3.6	3.4	7.8	6.9
Synthetic Missouri mean rate per 100 000 individuals after repeal	3.8	4.2	6.4	7.5
Estimated absolute change associated with PTP repeal^a^	−0.2	−0.8	1.4	−0.6
Estimated relative change associated with PTP repeal, %^b^	−5.2	−19.0	21.8	−8.1
States with change >Missouri, No.^c^				
<2 × Missouri RMSPE	1/3	0/6	0/6	3/11
**2011 concealed carry change**				
Missouri’s rate per 100 000 individuals after law change	3.8	4.3	8.3	8.4
Synthetic Missouri rate per 100 000 individuals after law change	4.0	4.2	9.6	8.4
Estimated absolute change associated with 2011 concealed carry law change^a^	−0.2	0.1	−1.3	0
Estimated relative change associated with 2011 concealed carry change, %^b^	−5.0	2.4	−13.5	−0.6
States with change >Missouri, No.^c^				
<2 × Missouri RMSPE	9/19	5/19	10/24	1/29
**2014 concealed carry change**				
Missouri’s rate per 100 000 individuals after law change	6.6	6.1	11.8	8.8
Synthetic Missouri rate per 100 000 individuals after law change	5.0	4.7	11.0	10.9
Estimated absolute change associated with 2014 concealed carry law change^a^	1.6	1.4	0.8	−2.1
Estimated relative change associated with 2014 concealed carry law change, %^b^	32.0	29.7	7.2	−19.2
States with change >Missouri, No.^c^				
<2 × Missouri RMSPE	1/15	0/12	2/33	6/29

Abbreviation: PTP, permit-to-purchase; RMSPE, root mean squared prediction error.

^a^ Mean difference between Missouri and synthetic Missouri in the postintervention period.

^b^ Percentage difference compared with synthetic Missouri.

^c^ Results from the permutation test, with associations computed as a difference in difference.

#### Concealed Carry 2011 Change

**Figure 2.**
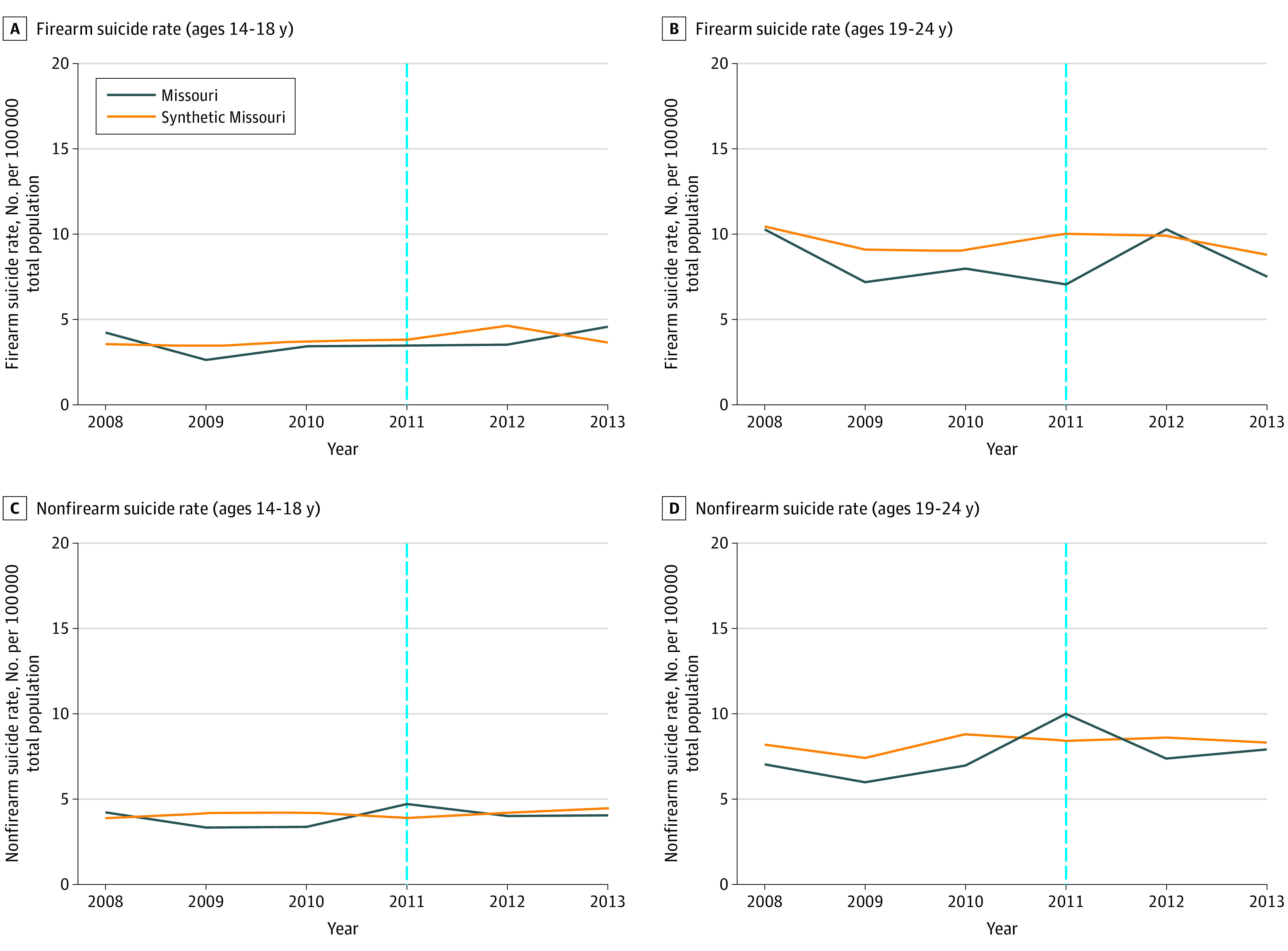
Trend in Annual Rate of Firearm and Nonfirearm Suicides per 100 000 People in Missouri and Synthetic Missouri Over the 2011 Concealed Carry Study Period The blue dotted line indicates the year the concealed carry law was passed in the Missouri state legislature. Synthetic Missouri is a weighted combination of control states that best approximated relevant characteristics of Missouri prior to the law changes examined by the study.

Of the 42 donor pool states, 39 had nonzero weights and were included in 1 or more of the synthetic controls for the 4 outcomes (eTables 2 and 3 in the Supplement). Trends for firearm suicide and nonfirearm suicide rates in the adolescent group in the preintervention period were similar for Missouri and synthetic Missouri (Figure 2). Trends for firearm suicide rates in the young adult group were similar in 2008, followed by increased rates in Missouri in the preintervention period (Figure 2). Trends in nonfirearm suicide rates in the young adult group were lower in Missouri in the preintervention period (Figure 2).

The Table shows the estimated absolute and relative associations between the 2011 concealed carry policy change and each outcome. The difference in firearm suicide rates between Missouri and synthetic Missouri in the adolescent group in the postintervention period was −0.2 per 100 000 people, corresponding to a 5.0% decrease. The difference in firearm suicide rates in the young adult group was −1.3 per 100 000 people, corresponding to a 13.5% decrease.

#### Concealed Carry 2014 Change

**Figure 3.**
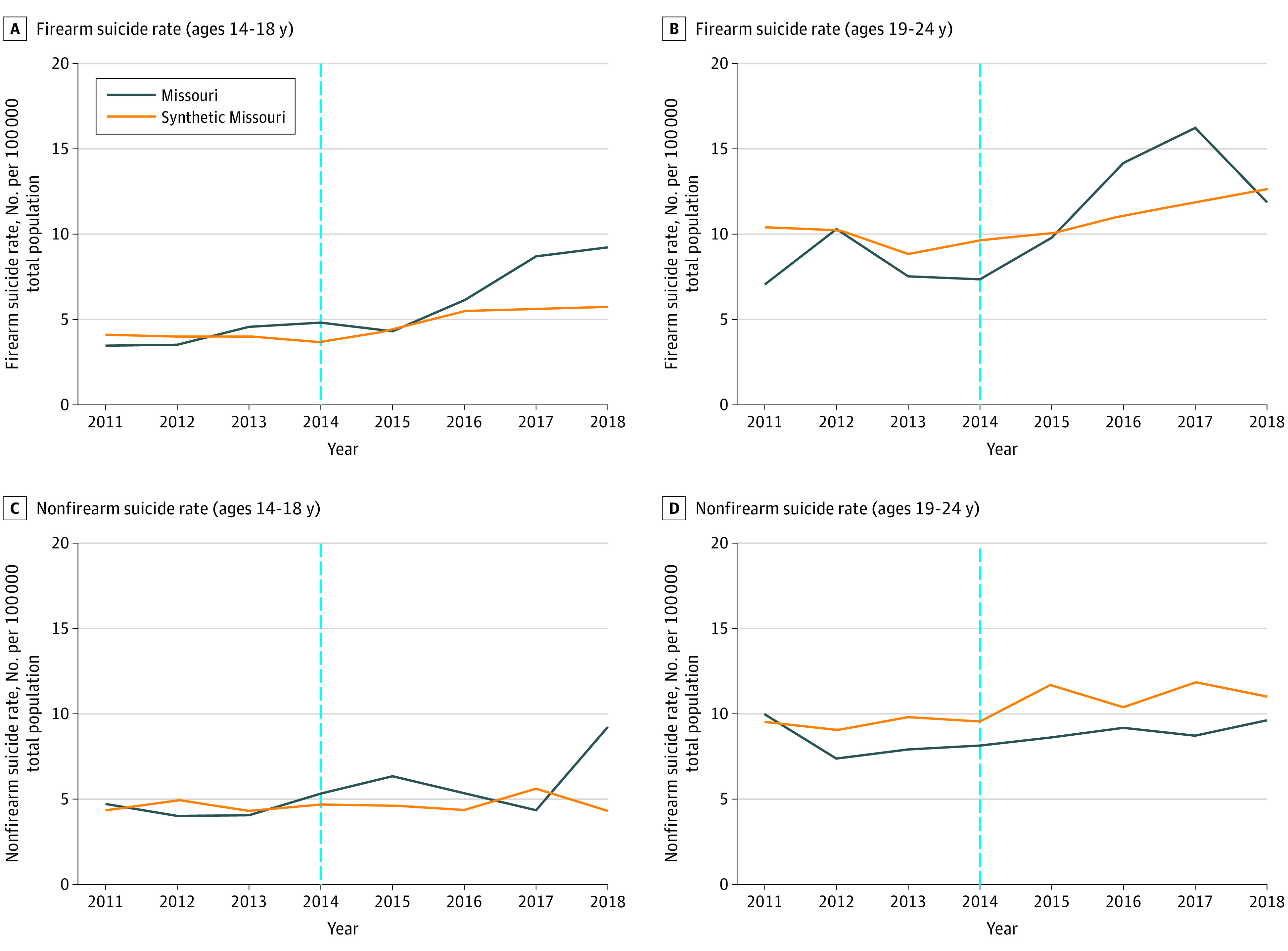
Trend in Annual Rate of Firearm and Nonfirearm Suicides per 100 000 People in Missouri and Synthetic Missouri Over the 2014 Concealed Carry Study Period The blue dotted line indicates the year the concealed carry law was passed in the Missouri state legislature. Synthetic Missouri is a weighted combination of control states that best approximated relevant characteristics of Missouri prior to the law changes examined by the study.

Of the 42 donor pool states, 39 had nonzero weights and were included in 1 or more of the synthetic controls for the 4 outcomes (the Supplement). Trends in firearm and nonfirearm suicide rates were similar in preintervention period in the youth group (Figure 3). Trends in firearm and nonfirearm suicide rates in the young adult group were lower in Missouri in the preintervention period (Figure 3).

The Table shows the estimated absolute and relative changes in suicide rates associated with Missouri’s 2014 change in Missouri’s concealed carry policy on each outcome. The mean difference in the rate of firearm suicides between Missouri and synthetic Missouri in the youth group in the postintervention period was 1.6 per 100 000 people, corresponding to a 32.0% increase, and a 29.7% increase was seen in nonfirearm suicide rates. The mean difference in firearm suicide rates in the young adult group was 0.8 per 100 000 people, corresponding to a 7.2% increase; a 19.2% decrease in nonfirearm suicide rates was seen.

## Discussion

This study evaluated the association between changes in Missouri’s PTP and concealed carry laws with firearm-related suicide rates in young Missouri residents. Our findings are consistent with previously published studies^31,33^ demonstrating increases in firearm suicides following Missouri’s PTP repeal in 2007. Firearm-related suicide rates after repeal of Missouri’s PTP law were 21.8% higher in the young adult group in Missouri, and nonfirearm suicide rates were 8.1% lower in Missouri. Previous studies^34,35^ showed Missouri’s PTP law repeal was also associated with increases in firearm-related homicides. Studies have shown links between gun ownership and youth suicide rates,^15^ that handgun ownership is associated with a greatly elevated and enduring risk of suicide by firearm,^36^ and that 82% of firearm-related suicides among young people involved a household member’s firearm.^15^ Repealing the PTP license could have increased access to firearms in those living with young, high-risk individuals who might have otherwise been screened out at their local sheriff's office. This may potentially explain the sudden increase in firearm suicide rates and reduction of nonfirearm suicide rates in the young adult group.

Firearm suicide rates after the 2011 concealed carry law change were lower for both age groups in Missouri, possibly because other confounding variables contributed to lowered suicide risk during that time (eg, heroin use, rates of depression, alcohol use, divorce rates). After the 2014 concealed carry law change, firearm suicide rates were 32.0% higher in the adolescent group in Missouri than in synthetic Missouri. However, nonfirearm suicide rates were also 29.7% higher in Missouri during that time period. This may be because of delayed effects of the 2011 firearm law change or the 2014 law change, or of other confounding variables contributing to overall increased suicide rates.

After the 2014 law change, firearm suicide rates in the young adult group were 7.2% higher, and nonfirearm suicide rates were 19.2% lower, which may suggest that young adults switched to firearm methods for suicide after this law change. It is important to note that under the 2007 Gun Control Act, shotguns or rifles may be sold only to individuals aged 18 years or older, and all other firearms may be sold only to individuals aged 21 years or older. It is possible that younger individuals were inspired to purchase firearms because they could now carry a concealed weapon, thus increasing the number of guns in circulation. For many individuals attempting suicide, the time between suicidal ideation and attempt can be as little as 10 minutes.^37^ Increased access to lethal means during periods of distress or impulsivity may unfortunately lead to a completed suicide in this age group.

### Limitations

This study had several limitations. We did not evaluate a 2017 law Missouri enacted that allowed for permitless carry in most public places^5,14^ because postintervention period data was only available until 2018. This could have affected analysis of the 2014 concealed carry law. For each state in the donor pool, we only evaluated for changes in a PTP or concealed carry law. We cannot be certain about other policies (related to firearms, public health, or mental health) that may have affected firearm suicides. We also did not include covariates in the young adult group related to depression or substance use, which may have affected suicide rates.

## Conclusions

Our findings show that changes in Missouri’s PTP law and concealed carry law were associated with increases in firearm suicide rates in young Missouri residents aged 14 to 24 years. Future studies are warranted to evaluate the impact of the 2017 concealed carry law change on suicide by firearms in this age group.

## Notes

Supplement. **eFigure 1.** Trends in Annual Rate of Firearm Suicide and Non-Firearm Suicide in Missouri and All Control States (PTP Change 2007) **eFigure 2.** Trends in Annual Rate of Firearm Suicide and Non-Firearm Suicide in Missouri and All Control States (Concealed Carry Change 2011) **eFigure 3.** Trends in Annual Rate of Firearm Suicide and Non-Firearm Suicide in Missouri and All Control States (Concealed Carry Change 2014) **eTable 1.** States With Nonzero Weights in Synthetic Missouri for Firearm and Non-Firearm Suicide Rates (PTP Repeal) **eTable 2.** States With Nonzero Weights in Synthetic Missouri for Firearm and Non-Firearm Suicide Rates (2011 Concealed Carry) **eTable 3.** States With Nonzero Weights in Synthetic Missouri for Firearm and Non-Firearm Suicide Rates (2014 Concealed Carry)
