# Supplementary material for: Association of Changes in Missouri Firearm Laws With Adolescent and Young Adult Suicides by Firearms
Source: JAMA Netw Open. 2020 Nov 4;3(11):e2024303. doi: 10.1001/jamanetworkopen.2020.24303 (PMC7643031; doi:10.1001/jamanetworkopen.2020.24303)
Supplement: Supplement. — eFigure 1. Trends in Annual Rate of Firearm Suicide and Non-Firearm Suicide in Missouri and All Control States (PTP Change 2007) eFigure 2. Trends in Annual Rate of Firearm Suicide and Non-Firearm Suicide in Missouri and All Control States (Concealed Carry Change 2011) eFigure 3. Trends in Annual Rate of Firearm Suicide and Non-Firearm Suicide in Missouri and All Control States (Concealed Carry Change 2014) eTable 1. States With Nonzero Weights in Synthetic Missouri for Firearm and Non-Firearm Suicide Rates (PTP Repeal) eTable 2. States With Nonzero Weights in Synthetic Missouri for Firearm and Non-Firearm Suicide Rates (2011 Concealed Carry) eTable 3. States With Nonzero Weights in Synthetic Missouri for Firearm and Non-Firearm Suicide Rates (2014 Concealed Carry) [file jamanetwopen-e2024303-s001.pdf]

Supplemental Online Content

Bhatt A, Wang X, Cheng A-L, et al. Association of changes in Missouri firearm laws with adolescent and young adult suicides by firearms. *JAMA Netw Open*. 2020;3(11):e2024303. doi: 10.1001/jamanetworkopen.2020.24303

eFigure 1. Trends in Annual Rate of Firearm Suicide and Non-Firearm Suicide in Missouri and All Control States (PTP Change 2007)
eFigure 2. Trends in Annual Rate of Firearm Suicide and Non-Firearm Suicide in Missouri and All Control States (Concealed Carry Change 2011)
eFigure 3. Trends in Annual Rate of Firearm Suicide and Non-Firearm Suicide in Missouri and All Control States (Concealed Carry Change 2014)
eTable 1. States With Nonzero Weights in Synthetic Missouri for Firearm and Non-Firearm Suicide Rates (PTP Repeal)
eTable 2. States With Nonzero Weights in Synthetic Missouri for Firearm and Non-Firearm Suicide Rates (2011 Concealed Carry)
eTable 3. States With Nonzero Weights in Synthetic Missouri for Firearm and Non-Firearm Suicide Rates (2014 Concealed Carry)

This supplemental material has been provided by the authors to give readers additional information about their work.

© 2020 Bhatt A et al. *JAMA Network Open.*

eFigure 1. Trends in annual rate of firearm suicide and non-firearm suicide in Missouri and all control states (PTP change 2007).

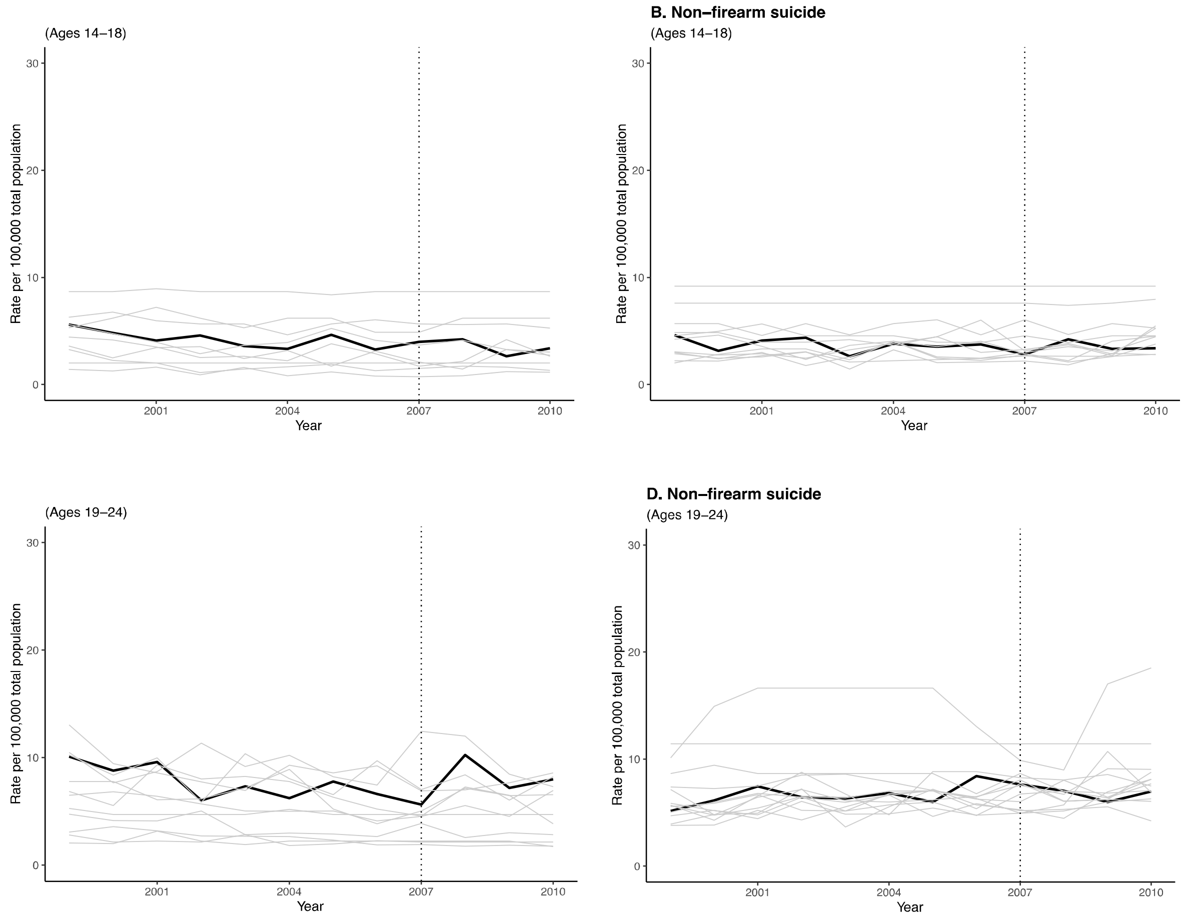

© 2020 Bhatt A et al. *JAMA Network Open.*

eFigure 2. Trends in annual rate of firearm suicide and non-firearm suicide in Missouri and all control states (Concealed Carry change 2011)

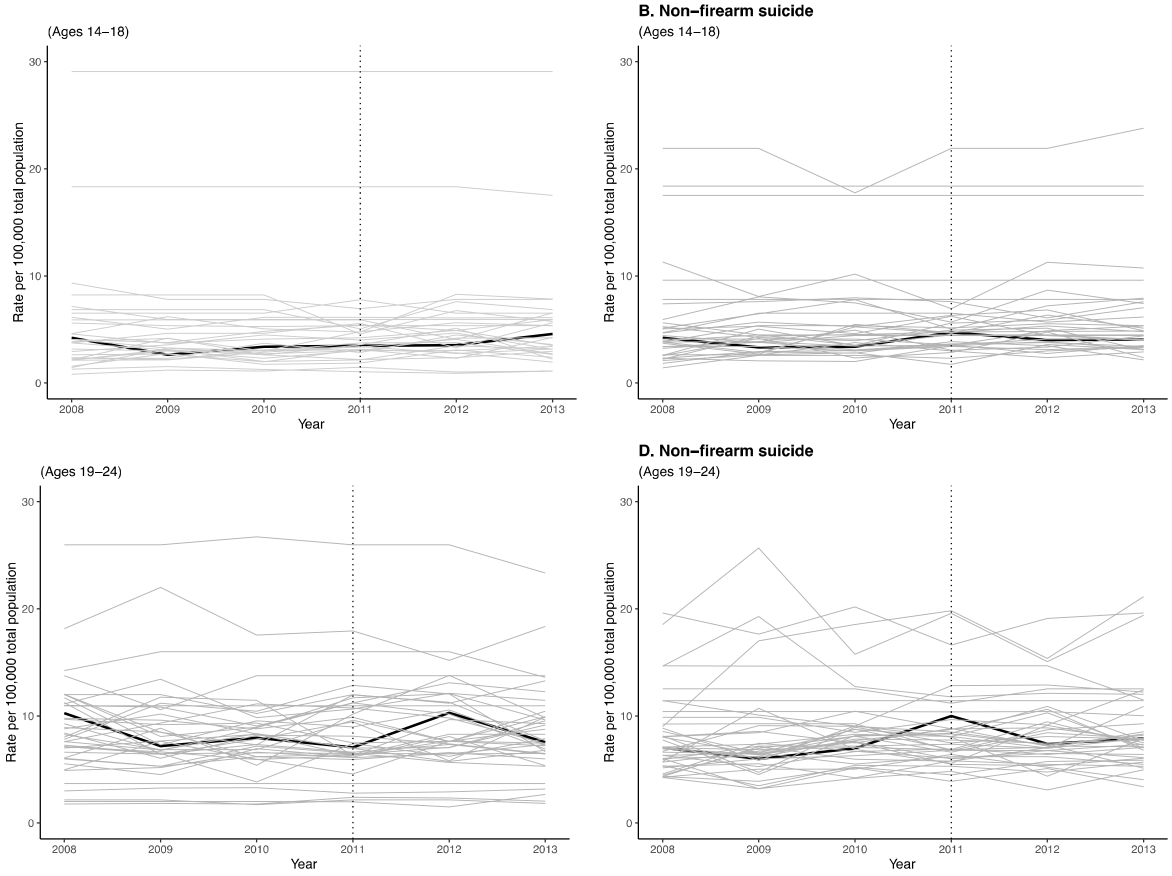

© 2020 Bhatt A et al. *JAMA Network Open.*

eFigure 3. Trends in annual rate of firearm suicide and non-firearm suicide in Missouri and all control states (Concealed Carry change 2014)

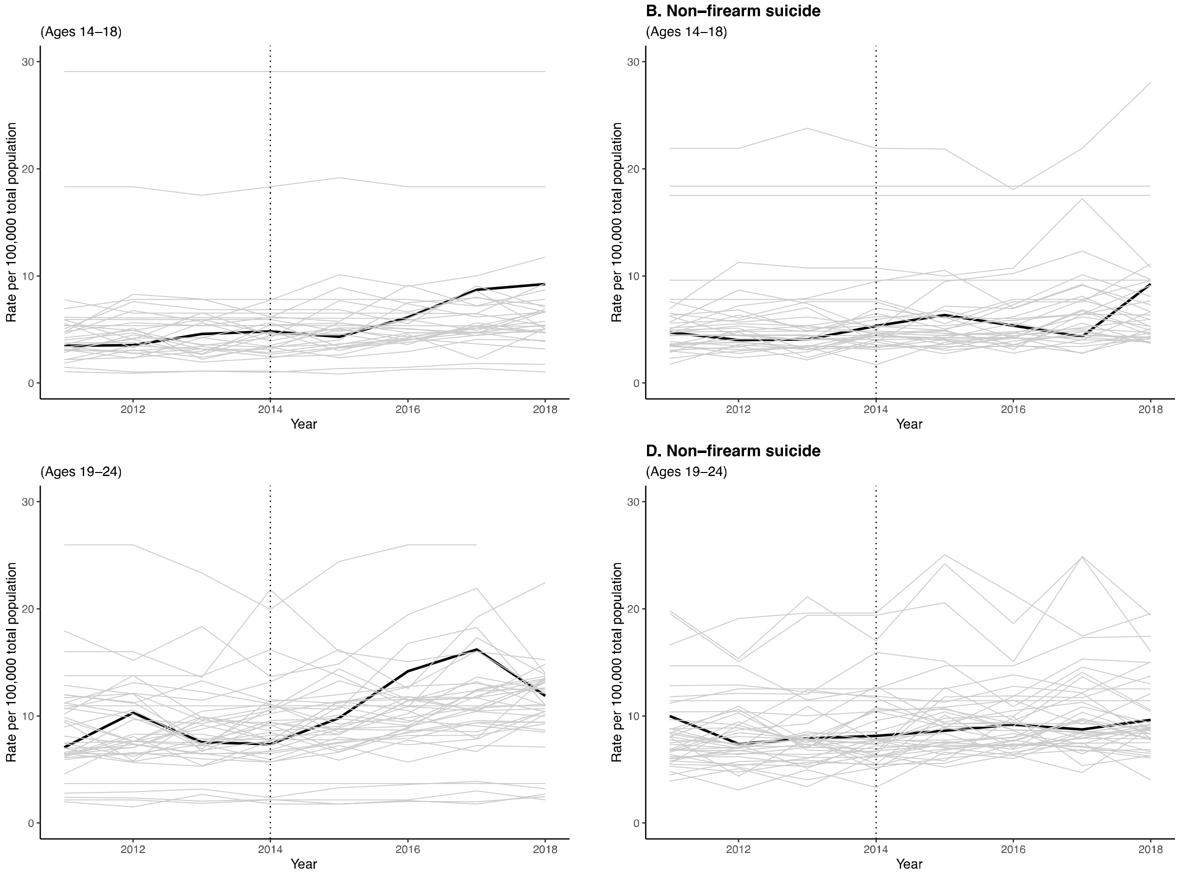

© 2020 Bhatt A et al. *JAMA Network Open.*

eTable 1. States with nonzero weights in synthetic Missouri for firearm and non-firearm suicide rates[a] (PTP repeal)

State	Firearm suicide Ages 14-18[b]	Firearm suicide Ages 19-24[b]	Non-firearm suicide Ages 14-18[c]	Non-firearm suicide Ages 19-24[c]
Connecticut	0	0	0	0.001
Hawaii	0	0	0	0.085
Illinois	0	0.001	0	0.054
Iowa	0.408	0.133	0.299	0.382
Kansas	0	0	0	0.001
Massachusetts	0	0	0.102	0.001
Michigan	0.132	0.420	0.599	0.474
Minnesota	0	0	0	0.001
Nebraska	0	0.445	0	0
New Jersey	0	0	0	0.001
New York	0.459	0	0	0

[a] States included in the donor pool (n = 13): Connecticut, Hawaii, Kansas, Illinois, Iowa, Massachusetts, Michigan, Minnesota, Nebraska, New Jersey, New York, North Carolina, and Rhode Island

[b] Covariates included in the 14-18-year-old models are rurality; unemployment rates; percent below poverty; educational attainment; household gun ownership; prevalence of severe negative affect; seriously considered attempting suicide; suicidal planning; suicide attempts; marijuana use; heroin use.

[c] Covariates included in the 19-24-year-old models are rurality; unemployment rates; percent below poverty; educational attainment; household gun ownership.

© 2020 Bhatt A et al. *JAMA Network Open.*

eTable 2. States with nonzero weights in synthetic Missouri for firearm and non-firearm suicide rates[a] (2011 Concealed Carry)

**Ages 14-18 Firearm [b]**		**Ages 14-18 Non-firearm [b]**		**Ages 19-24 Firearm [c]**		**Ages 19-24 Non-firearm [d]**	
0.005	ALASKA	0.001	ALASKA	0.007	ALABAMA	0.003	ALABAMA
0.22	ARIZONA	0.173	ARIZONA	0.005	ALASKA	0.017	ARIZONA
0.001	ARKANSAS	0.254	INDIANA	0.017	ARIZONA	0.02	ARKANSAS
0.197	INDIANA	0.061	KENTUCKY	0.043	ARKANSAS	0.01	CALIFORNIA
0.134	KANSAS	0.004	MICHIGAN	0.012	CALIFORNIA	0.019	COLORADO
0.01	KENTUCKY	0.379	PENNSYLVANIA	0.016	COLORADO	0.008	CONNECTICUT
0.029	MICHIGAN	0.064	SOUTH CAROLINA	0.008	CONNECTICUT	0.009	FLORIDA
0.008	MISSISSIPPI	0.064	TENNESSEE	0.011	FLORIDA	0.009	GEORGIA
0.003	OKLAHOMA			0.012	GEORGIA	0.015	HAWAII
0.211	PENNSYLVANIA			0.015	HAWAII	0.009	INDIANA
				0.013	INDIANA	0.019	IOWA
				0.02	IOWA	0.02	KANSAS
				0.019	KANSAS	0.016	LOUISIANA
				0.012	KENTUCKY	0.017	MAINE
				0.018	LOUISIANA	0.007	MASSACHUSETTS
				0.017	MAINE	0.001	MICHIGAN
				0.008	MASSACHUSETTS	0.015	MINNESOTA
				0.012	MICHIGAN	0.077	MISSISSIPPI
				0.016	MINNESOTA	0.022	MONTANA
				0.052	MISSISSIPPI	0.06	NEBRASKA
				0.022	MONTANA	0.378	NEVADA
				0.022	NEBRASKA	0.011	NEW HAMPSHIRE
				0.334	NEVADA	0.006	NEW JERSEY
				0.014	NEW HAMPSHIRE	0.034	NEW MEXICO
				0.006	NEW JERSEY	0.012	NEW YORK
				0.066	NEW MEXICO	0.01	NORTH CAROLINA
				0.011	NEW YORK	0.01	OHIO
				0.012	NORTH CAROLINA	0.025	OKLAHOMA
				0.012	OHIO	0.014	OREGON
				0.025	OKLAHOMA	0.011	PENNSYLVANIA
				0.015	OREGON	0.007	RHODE ISLAND
				0.012	PENNSYLVANIA	0.008	SOUTH CAROLINA
				0.012	SOUTH CAROLINA	0.029	SOUTH DAKOTA
				0.026	SOUTH DAKOTA	0.008	TENNESSEE
				0.013	TENNESSEE	0.019	TEXAS
				0.017	TEXAS	0.017	UTAH

© 2020 Bhatt A et al. *JAMA Network Open.*

				0.018	UTAH	0.014	WASHINGTON
				0.014	WASHINGTON	0.014	WEST VIRGINIA
				0.014	WYOMING		

[a] States included in the donor pool (n = 42): Alabama, Alaska, Arizona, Arkansas, California, Colorado, Connecticut, Delaware, Florida, Georgia, Hawaii, Indiana, Iowa, Kansas, Kentucky, Louisiana, Maine, Massachusetts, Michigan, Minnesota, Mississippi, Montana, Nebraska, Nevada, New Hampshire, New Jersey, New Mexico, New York, North Carolina, Ohio, Oklahoma, Oregon, Pennsylvania, Rhode Island, South Carolina, South Dakota, Tennessee, Texas, Utah, Washington, West Virginia, Wyoming.

[b] Covariates included in the 14-18-year-old models are rurality; unemployment rates; percent below poverty; educational attainment; household gun ownership; prevalence of severe negative affect; seriously considered attempting suicide; suicidal planning; suicide attempts.

[c] Covariates included in the 19-24-year-old models are rurality; unemployment rates; percent below poverty; educational attainment; household gun ownership.

© 2020 Bhatt A et al. *JAMA Network Open.*

eTable 3. States with nonzero weights in synthetic Missouri for firearm and non-firearm suicide rates [a] (2014 Concealed Carry)

**Ages 14-18 Firearm [b]**		**Ages 14-18 Non-firearm [b]**		**Ages 19-24 Firearm [c]**		**Ages 19-24 Non-firearm [d]**	
0.245	IOWA	0.002	COLORADO	0.024	ALABAMA	0.019	ALABAMA
0.001	KANSAS	0.106	KENTUCKY	0.022	ALASKA	0.092	ALASKA
0.002	NEW MEXICO	0.182	MASSACHUSETTS	0.019	ARIZONA	0.017	ARIZONA
0.272	OHIO	0.285	OKLAHOMA	0.024	ARKANSAS	0.234	ARKANSAS
0.204	SOUTH CAROLINA	0.195	SOUTH CAROLINA	0.017	CALIFORNIA	0.009	CALIFORNIA
0.277	TENNESSEE	0.231	TENNESSEE	0.017	COLORADO	0.015	COLORADO
0.245	IOWA			0.017	CONNECTICUT	0.007	CONNECTICUT
0.001	KANSAS	0.002	COLORADO	0.02	FLORIDA	0.008	FLORIDA
0.002	NEW MEXICO	0.106	KENTUCKY	0.018	GEORGIA	0.01	GEORGIA
0.272	OHIO	0.182	MASSACHUSETTS	0.021	HAWAII	0.014	HAWAII
				0.023	INDIANA	0.015	INDIANA
				0.023	IOWA	0.027	IOWA
				0.021	KANSAS	0.028	KANSAS
				0.025	KENTUCKY	0.017	KENTUCKY
				0.021	MAINE	0.019	LOUISIANA
				0.017	MASSACHUSETTS	0.025	MAINE
				0.019	MICHIGAN	0.005	MASSACHUSETTS
				0.02	MINNESOTA	0.011	MICHIGAN
				0.021	MONTANA	0.019	MINNESOTA
				0.023	NEBRASKA	0.041	MONTANA
				0.021	NEVADA	0.031	NEBRASKA
				0.02	NEW HAMPSHIRE	0.073	NEVADA
				0.018	NEW JERSEY	0.018	NEW HAMPSHIRE
				0.014	NEW MEXICO	0.005	NEW JERSEY
				0.017	NEW YORK	0.02	NEW MEXICO
				0.018	NORTH CAROLINA	0.006	NEW YORK
				0.022	OHIO	0.01	NORTH CAROLINA
				0.027	OKLAHOMA	0.01	OHIO
				0.018	OREGON	0.037	OKLAHOMA
				0.021	PENNSYLVANIA	0.018	OREGON
				0.02	SOUTH CAROLINA	0.012	PENNSYLVANIA
				0.024	SOUTH DAKOTA	0.005	RHODE ISLAND
				0.024	TENNESSEE	0.011	SOUTH CAROLINA
				0.022	TEXAS	0.038	SOUTH DAKOTA
				0.021	UTAH	0.014	TENNESSEE
				0.018	WASHINGTON	0.017	TEXAS

© 2020 Bhatt A et al. *JAMA Network Open.*

				0.237	WEST VIRGINIA	0.026	UTAH
				0.024	WYOMING	0.015	WASHINGTON
						0.003	WEST VIRGINIA

[a] States included in the donor pool (n = 42): Alabama, Alaska, Arizona, Arkansas, California, Colorado, Connecticut, Delaware, Florida, Georgia, Hawaii, Indiana, Iowa, Kansas, Kentucky, Louisiana, Maine, Massachusetts, Michigan, Minnesota, Mississippi, Montana, Nebraska, Nevada, New Hampshire, New Jersey, New Mexico, New York, North Carolina, Ohio, Oklahoma, Oregon, Pennsylvania, Rhode Island, South Carolina, South Dakota, Tennessee, Texas, Utah, Washington, West Virginia, Wyoming.

[b] Covariates included in the 14-18-year-old models are rurality; unemployment rates; percent below poverty; educational attainment; household gun ownership; prevalence of severe negative affect; seriously considered attempting suicide; suicidal planning; suicide attempts.

[c] Covariates included in the 19-24-year-old models are rurality; unemployment rates; percent below poverty; educational attainment; household gun ownership.

© 2020 Bhatt A et al. *JAMA Network Open.*
